# Screening cultures for detection of methicillin-resistant *Staphylococcus aureus* in a population at high risk for MRSA colonisation: identification of optimal combinations of anatomical sites

**DOI:** 10.3402/ljm.v8i0.22755

**Published:** 2013-11-26

**Authors:** Khalid El-Bouri, Wahbi El-Bouri

**Affiliations:** 1 Infection Prevention and Control Department, Singleton Hospital, Abertawe Bro-Morgannwg University Hospital Board, Swansea, UK; 2 Institute of Biomedical Engineering, University of Oxford, Headington, Oxford, UK

**Keywords:** MRSA screening, optimal sensitivity, infection control

## Abstract

This retrospective study analysed the diagnostic yield of single-site, two-site, and three-site anatomical surveillance cultures in a population of 4,769 patients at high risk for methicillin-resistant *Staphylococcus aureus* (MRSA) colonisation. Cultures of seven anatomical sites were used as the gold standard against which to measure the sensitivity of MRSA detection. Detection rates for the seven single-sites, 21 two-site, and 35 three-site combinations are presented. Single-site swabbing only detected 50.5% (nose) of total cases, while three-site surveillance achieved a 92% (groin + nose + throat) sensitivity of detection at best. It is recommended that at least three anatomical sites should be screened for MRSA colonisation in these high-risk patients.

Infections arising from methicillin-resistant *Staphylococcus aureus* (MRSA) are an important cause of healthcare-associated infections (HCAI) throughout the world. They are associated with an increase in morbidity, mortality, and an increase in healthcare costs and there are indications that MRSA infections are increasing worldwide (1). Individuals colonised with MRSA are at a greater risk of developing infections with this organism (2). The aim of this retrospective study was to determine the optimal body sites and combinations of sites for detection of MRSA carriage in a high-risk population using sampling from seven anatomical sites as a gold standard. Single-site swabbing has a low sensitivity of detection despite it being recommended in some guidelines (3). Using culture on selective media, we determined the sensitivity of a single anatomical site sampling in this high-risk population as well as all possible combinations of two and three anatomical sites to find the highest detection rates.

## Materials and methods

Between January 1 2010 and November 30 2012, 4,769 sets of body swabs were taken from adult patients admitted to the Abertawe Bro-Morgannwg University Hospital in Swansea, Wales. The body sites swabbed in all patients were as follows: axilla, hairline, groin, nose, perineum, throat, and umbilicus. Only patients where all these anatomical sites were swabbed simultaneously were accepted to this retrospective study. These were adult patients who were classed as being at high risk for MRSA colonisation because of their frequent re-admissions to healthcare facilities; direct inter-hospital transfers; recent admissions at a hospital known or likely to have a high prevalence of MRSA; residents of nursing or residential care homes; patients being admitted to high-risk areas; transfers from outside the United Kingdom, or patients with a history of colonisation with MRSA in the past. Upon admission, swabs were taken from the above-mentioned body sites, transported in charcoal transport medium (Amies) and plated separately onto chromogenic MRSA medium (P&O Laboratories). Suspect colonies were subcultured onto Columbia blood agar (Oxoid Ltd) and identified with a latex agglutination kit (Pro-Lab) and each new isolate further tested on panel PMIC/ID-67 of the Becton-Dickenson Phoenix ID system for confirmation of identity and provision of an antimicrobial susceptibility profile. *S. aureus* isolates were also identified using mass spectrometry, MALDI-TOF (Bruker) (4, 5). Patients were considered colonised if MRSA was grown from any of the body sites tested. The seven-site body screen was regarded as the reference standard against which the sensitivity of single and combined swabs was measured.

### Statistics

The number of different combinations for the two-site and the three-site determinations were calculated to be seven for the single-site swabs, 21 for the two-site swabs, and 35 for the three-site swabs. Confidence intervals were calculated using the normal approximation method of the binomial confidence interval.

Ethical approval was deemed unnecessary by the local ethics committee, as this was a retrospective study.

## Results

The number of seven-site anatomical sampling screens taken from 4,769 individuals that detected MRSA was 925 (19.4%). The patient was considered colonised with MRSA if any one of the swabs was found positive for MRSA. Single-site swabs detected MRSA colonisation at rates between 18% (hairline) and 50.5% (nose) as shown in Table 1. The ability of two anatomical site swabs to improve the detection of MRSA colonisation was determined by using all possible combinations from the original set of seven sites sampled. There were 21 combinations possible and these are shown in Table 1. The two-site swabs improved the detection rate of MRSA by up to 50% with the best combination being the groin and throat where 74.5% of colonisations were detected. These were closely followed by the groin/nose, nose/perineum, and the nose/throat combinations. Of note is that some of the two-swab combinations, such as the axilla/hairline and the hairline/umbilicus combinations, had poorer detection rates than single swabs of the nose, groin, perineum, or throat. However, 14 other two-site combinations were more sensitive at detecting MRSA colonisation than the single nasal swab (Table 1).

**Table:** Numbers of MRSA colonisations detected at different anatomical sites and combinations of two and three sites

Anatomical site	No. of patients MRSA detected (total = 925)	Sensitivity (%) of detection (95% CI)
**Single-site**		
Nose	467	50.5% (47.3–53.7)
Throat	444	48% (44.8–51.2)
Groin	408	44.2% (40.9–47.3)
Perineum	365	39.5% (36.3–42.6)
Umbilicus	214	23.1% (20.4–25.9)
Axilla	171	18.5% (16–21)
Hairline	167	18% (15.6–20.5)
**Two-site**		
Groin + throat	689	74.5% (71.7–77.3)
Groin + nose	667	72.1% (69.2–75)
Nose + perineum	664	71.8% (68.9–74.7)
Nose + throat	662	71.6% (68.7–74.5)
Perineum + throat	582	63% (59.8–66)
Throat + umbilicus	553	59.8% (56.6–62.9)
Nose + umbilicus	549	59.35% (56.2–62.5)
Axilla + nose	524	56.6% (53.5–59.8)
Axilla + throat	521	56.3% (53.1–59.5)
Groin + perineum	518	56% (52.8–59.2)
Hairline + throat	513	55.46% (52.3–58.7)
Hairline + nose	501	54.1% (51–57.4)
Groin + umbilicus	482	52.1% (48.9–55.3)
Groin + hairline	476	51.45% (48.2–54.7)
Axilla + groin	457	49.4% (46.2–52.6)
Perineum + umbilicus	455	49.2% (46–52.4)
Axilla + perineum	435	47% (43.8–50.2)
Hairline + perineum	432	46.7% (43.5–49.9)
Axilla + umbilicus	300	32.4% (29.4–35.4)
Hairline + umbilicus	293	31.7% (28.7–34.7)
Axilla + hairline	267	28.8% (25.9–31.8)
**Three-site**		
Groin + nose + throat	850	92% (90.1–93.6)
Nose + perineum + throat	840	91% (88.9–92.7)
Groin + perineum + throat	783	84.6% (82.3–87)
Groin + nose + perineum	769	83.1% (80.7–85.5)
Groin + throat + umbilicus	752	81.3% (78.8–83.8)
Nose + throat + umbilicus	742	80.2% (77.6–82.8)
Axilla + groin + throat	732	79.1% (76.5–81.7)
Groin + hairline + throat	726	78.5% (75.8–81.1)
Groin + nose + umbilicus	724	78.3% (75.6–81)
Perineum + throat + umbilicus	720	77.8% (75.2–80.5)
Nose + perineum + umbilicus	719	77.7% (75–80.4)
Axilla + nose + throat	717	77.5% (74.8–80.2)
Groin + hairline + nose	708	76.5% (73.8–79.3)
Hairline+ perineum + throat	706	76.3% (73.6–79)
Axilla + perineum +throat	703	76% (73.2–78.7)
Hairline + nose + perineum	698	75.5% (72.7–78.2)
Axilla + nose + perineum	694	75% (72.2–77.8)
Axilla + groin + nose	693	74.9% (72.1–77.7)
Hairline + nose + throat	690	74.6% (71.8–77.4)
Axilla + nose + umbilicus	604	65.3% (62.2–68.4)
Axilla + throat + umbilicus	597	64.5% (61.5–67.6)
Hairline + throat + umbilicus	596	64.4% (61.3–67.5)
Groin + perineum + umbilicus	583	63% (59.9–66.1)
Groin + hairline + perineum	582	62.9% (59.8–66)
Axilla + hairline + throat	581	62.8% (59.7–65.9)
Hairline + nose + umbilicus	581	62.8% (59.7–65.9)
Axilla + groin + perineum	577	62.4% (59.2–65.5)
Groin + hairline + umbilicus	543	58.7% (55.5–61.9)
Axilla + hairline + nose	535	57.8% (54.6–61)
Axilla + groin + umbilicus	534	57.7% (54.5–60.9)
Axilla + groin + hairline	522	56.4% (53.2–59.6)
Axilla + perineum + umbilicus	516	55.8% (52.6–59)
Hairline + perineum + umbilicus	506	54.7% (51.5–57.9)
Axilla + hairline + perineum	481	52% (48.8–55.2)
Axilla + hairline + umbilicus	367	39.6% (36.5–42.8)

There were 925 patients found to be colonised with MRSA in total using the seven-site body screen gold standard. CI, confidence interval; MRSA, methicillin-resistant *Staphylococcus aureus*.

Three anatomical site combinations yielded even better results than the two-site anatomical combinations when compared to the seven-site anatomical gold standard. Table 1 shows all 35 different combinations of three anatomical site sampling with the best detection rate accounted for by a combination of groin + nose + throat, which detected 92% of colonisations closely followed by perineum + nose + throat combinations (91%). It can be noted from Table 1 that all but one of the triple-site sampling had detection rates superior to that of the single nose swab. The only combination with inferior results was the axilla + hairline + umbilicus combination. Sampling of the groin and throat together improved MRSA detection by 47% and sampling of the groin, nose and throat together improved MRSA detection by 82% when compared to single-site sampling from the nose.

## Discussion

In this study, we assessed the role of MRSA screening in patients at high risk for MRSA colonisation. Sampling from seven anatomic body sites was used as the reference gold standard against which to measure single-site, two-site, and three-site sampling combinations. This was to determine the smallest combination of surface body sites that can give the highest detection rates of MRSA colonisation. Colonisation with MRSA is associated with an increased likelihood of clinical infection with MRSA (6). Screening patients for MRSA has been advocated to detect carriers and enforce decolonisation procedures which can reduce infections and prevent transmission to others. Different countries have differing policies on the screening procedures. US guidelines suggest only that nasal swabbing may be sufficient (3), whereas many in the United Kingdom recommend at least two sites for screening (7, 8). In areas of low endemicity, single-site screening may be sufficient to detect colonisation. However, in patients at high risk for MRSA colonisation, the single-site nasal swab is likely to miss a significant number of colonisations and allow spread of the organism in healthcare establishments hence putting patients at risk of developing infections with MRSA. In this study, we found that swabs of the anterior nares alone detected only about half (50.5%) of all possible cases whereas a triple swab combination of nose, groin, and throat detected 92% of colonisations. This contrasts with the Scottish results where swabs of the nares detected 66.4% of MRSA colonisations (9). Throat swabs alone performed less well (10). Table 1 shows the results of two-site swabbing, whereby groin and throat combinations improved sensitivity of detection to 74.5%. It can also be seen that 10 of the 21 two-site combinations gave superior results to the nose swab alone. Certain anatomical site combinations lacked sensitivity and gave poor results. Axilla, hairline, and umbilicus combinations could not achieve the sensitivity of single nose or throat swabs (Table 1). However, triple swab combinations of most sites were as sensitive as or of higher sensitivity than nasal, throat, or groin swabs individually. From Table 1, it can be seen that only one of the 35 different combinations failed to achieve the sensitivity of the single nasal swab. Optimal two-site screening is likely to miss up to 25% of MRSA colonisations. This study is unique in that it calculates all of the different combinations of anatomic sites including all 21 possible two-site and all 35 three-site combinations. This allows clinicians to predict the relative accuracy of combination swab results in relation to the commonly used nasal swab. This may be useful where nasal, throat, or perineum swabs cannot be taken for medical or personal reasons. In addition, the numbers of patients in this study are relatively high. It would be useful to have further studies, assessing all seven anatomical sites, similar to this one in high-risk patients using rapid molecular methodologies. These molecular methods can be commercial or in-house (11–13). This would allow a faster turn-around time of results and allow appropriate decolonisation procedures and infection control measures to be implemented more rapidly. Although there have been studies showing the merits of universal decolonisation in intensive care units, these cannot be used for all areas of healthcare and it remains important to screen for MRSA in most institutions because of the risks of infection and transmission (14–19). Because screening is relatively expensive, it is important to determine the optimal and most cost-effective combination of anatomical sites that are tested. Our results indicate that a three-site screen, preferably including groin, nose, and throat will detect the vast majority of cases. The sensitivity of all other possible combinations is also presented. We feel that the results of this study will help healthcare managers to decide the optimal strategy that can be adopted to optimise the ability to detect MRSA colonisation and prevent infection and nosocomial transmission in this at-risk population.

## References

[1] David MZ, Daum RS (2010). Community-associated methicillin-resistant *Staphylococcus aureus*: epidemiology and clinical consequences of an emerging epidemic. Clin Microbiol Rev.

[2] Reilly JS, Stewart S, Christie P, Allardice GM, Stari T, Matheson A (2012). Universal screening for methicillin-resistant *Staphylococcus aureus* in acute care: risk factors and outcome from a multicentre study. J Hosp Inf.

[3] Jernigan J, Kallen A (2010). Methicillin-resistant *Staphylococcus aureus* (MRSA) infections, activity C: ELC prevention collaboratives. http://www.cdc.gov/HAI/pdfs/toolkits/MRSA_toolkit_white_020910_v2.pdf.

[4] El-Bouri K, Johnston S, Rees E, Thomas I, Bome-Mannothoko N, Jones C (2012). Comparison of bacterial identification by MALDI-TOF mass spectrometry and conventional diagnostic microbiology methods: agreement, speed and cost implications. Br J Biomed Sci.

[5] Harris LG, El-Bouri K, Johnston S, Rees E, Frommelt L, Siemssen N (2010). Rapid identification of staphylococci from prosthetic joint infections using MALDI-TOF mass-spectrometry. Int J Artif Organs.

[6] Davis KA, Stewart JJ, Crouch HK, Florez CE, Hospenthal DR (2004). Methicillin-resistant *Staphylococcus aureus* (MRSA) nares colonisation at hospital admission and its effect on subsequent MRSA infection. Clin Inf Dis.

[7] NHS Scotland MRSA Screening Pathfinder Programme (2010). http://www.documents.hps.scot.nhs.uk/hai/mrsa-screening/pathfinder-programme/mrsa-pathfinder-vol1–2011-02-23.pdf.

[8] McBride M (2008). Best practice on screening for methicillin-resistant *Staphylococcus aureus* (MRSA) colonisation. http://www.dhsspsni.gov.uk/hss-md-12-2008.pdf.

[9] Matheson A, Christie P, Stari T, Kavanagh K, Gould IM, Masterton R (2012). Nasal swab screening for methicillin-resistant Staphylococcus aureus—how well does it perform? A cross-sectional study. Infect Control Hosp Epidemiol.

[10] Harbarth S, Schrenzel J, Renzi G, Akakpo C, Ricou B (2007). Is throat screening necessary to detect methicillin-resistant Staphylococcus aureus colonisation in patients upon admission to an intensive care unit?. J Clin Microbiol.

[11] Jukes L, Mikhail J, Bome-Mannathoko N, Hadfield SJ, Harris LG, El-Bouri K (2010). Rapid differentiation of *Staphylococcus aureus*, *Staphylococcus epidermidis*, and other coagulase-negative staphylococci and methicillin susceptibility testing directly from growth-positive blood cultures by multiplex real-time PCR. J Med Microbiol.

[12] Wassenberg MWM, Kluytsmans JAJW, Bosboom RW, Buiting AGM, van Elzakker EPM, Melchers WJG (2011). Rapid diagnostic testing of methicillin-resistant *Staphylococcus aureus* carriage at different anatomical sites: costs and benefits of less extensive screening regimens. CMI.

[13] Senn L, Basset P, Nahimana I, Zanetti G, Blanc DS (2011). Which anatomical sites should be sampled for screening of methicillin-resistant *Staphylococcus aureus* carriage by culture or by rapid PCR test?. CMI.

[14] Huang SS, Septimus E, Kleinman K, Moody J, Hicock J, Avery TR (2013). Targeted versus universal decolonisation to prevent ICU infections. N Engl J Med.

[15] Huang SS, Yokoe DS, Hinrichsen VL, Spurchise LS, Datta R, Miroshnik I (2006). Impact of routine intensive care surveillance cultures and resultant barrier precautions on hospital-wide methicillin-resistant *Staphylococcus aureus* bacteraemia. Clin Inf Dis.

[16] Cosgrove SE, Sakoulas G, Perencevich EN, Schwaber MJ, Karchmer AW, Carmeli Y (2003). Comparison of mortality associated with methicillin-resistant and methicillin-susceptible *Staphylococcus aureus* Bacteraemia: a meta-analysis. Clin Inf Dis.

[17] Lautenbach E, Nachamkin I, Hu B, Fishman NO, Tolomeo P, Prasad P (2009). Surveillance cultures for detection of methicillin-resistant *Staphylococcus aureus*: diagnostic yield of anatomic sites and comparison of provider- and patient-collected samples. Infect Control Hosp Epidemiol.

[18] Syndor ERM, Perl T (2011). Hospital epidemiology and infection control in acute care settings. Clin Microbiol Rev.

[19] Haley CC, Mittal D, LaVioletta A, Jannapureddy S, Parvez N, Haley RW (2007). Methicillin-resistant *Staphylococcus aureus* infection or colonisation present at hospital admission: multivariable risk factor screening to increase efficiency of surveillance culturing. J Clin Microbiol.

